# Supplementary material for: Graphical analysis of guideline adherence to detect systemwide anomalies in HIV diagnostic testing
Source: PLoS One. 2022 Jul 1;17(7):e0270394. doi: 10.1371/journal.pone.0270394 (PMC9249187; doi:10.1371/journal.pone.0270394)
Supplement: S1 File — (DOCX) [file pone.0270394.s001.docx]

Graphical Analysis of Guideline Adherence to Detect Systemwide Anomalies in HIV Diagnostic Testing

Supplement

1. Consideration of Absolute Time (Text)
2. Example 1: Modeling Guideline Adherence with Graphical Techniques
   1. Figures S1-S3
   2. Tables S1-S3
3. Example 2: Consideration of Absolute Time
   1. Figures S4-S6
   2. Tables S4-S7
4. Count of Possible Edges in a Graph
5. Supplementary Tables & Figures
   1. Table S8: Description of HIV Tests
   2. Figure S7. Data flow from Input (i.e., Observational Medical Database) to Output (i.e., Graphs, Reports)
   3. Figure S8. Modeling of HIV Diagnostic Testing Guidelines as a Graph - Alternative to Figure 1

Consideration of Absolute Time

To construct a clinically meaningful model of guideline adherence with a directed graph, an important consideration is absolute time, in addition to the chronological sequence of tests (e.g., test 1 🡪 test 2). By absolute time we mean the elapsed time measured in, for example, seconds or minutes, between one test and another. We modified the graphical model of guideline adherence to account for absolute time and improve its clinical interpretation.

If two events occur at the exact same time, their order in a chronologic sequence sorted by time alone becomes ambiguous. A blind assumption that one test occurs before another in the sequence could lead to guideline nonadherence, where the reversed sequence order would not. Certain facilities within our healthcare system have workflows that actualize this theoretical consideration. First, “5^th^ generation” HIV tests perform the HIV screen and confirmation together in a single test. The results of these two tests may then occur at exactly the same time. Second, reference laboratories contracted to perform HIV diagnostic testing reported the results of both the HIV screen and, if necessary, the confirmation at the same time, even if they performed them sequentially. Third, other facilities delayed the release of a positive HIV screen until the confirmation test, to avoid the potential for misinterpretation of a positive HIV screen without confirmation as diagnostic of HIV. They release both results at the same time.

When two events can occur at the same time, they also frequently occur with either event preceding the other. The time elapsed between events may equate to a fraction of a second, but for a chronological ordering, the difference in absolute time does not matter. As an example, a “5^th^ generation” HIV test may report the HIV confirmation milliseconds before the HIV screen. Although performed together, the chronological sequence of tests resembles guideline nonadherence because the HIV confirmation should not precede the screen.

The format used by a clinical laboratory to report the result of a test can also complicate the temporal relationship and, consequently, its interpretation in a directed graph. For example, clinical laboratories commonly report HIV confirmation tests as two separate results (e.g., HIV-1 and HIV-2). While clinically equivalent to a single HIV confirmation test, they represent two HIV confirmation tests in the directed graph so far described. An HIV confirmation test followed by a second HIV confirmation test represents guideline nonadherence, despite their close temporal proximity.

To account for absolute time in the model, we grouped together tests that occurred within a time window of seven days. Tests grouped together became new nodes in the graph. For example, two HIV confirmation tests reported at the same time (e.g. HIV-1 confirmation, positive and HIV-2 confirmation, negative) would become a new node, “+Confirm, -Confirm”. Generation of new nodes while modeling patient HIV testing as a graph also required consideration of the new nodes in the context of the graph representing the HIV Diagnostic Testing Guidelines. For example, we added the new node to the graph of HIV Diagnostic Testing Guidelines and created an edge to “+Confirm, -Confirm” from a positive screen. The creation of new nodes and edges allowed the graphical model to account for absolute time.

## Example 1: Modeling Guideline Adherence with Graphical Techniques

This fictitious example demonstrates a method to assess guideline adherence with graphical techniques. The computer code for these examples is available.^16^

1. Construct the expected graph based on the problem domain

| 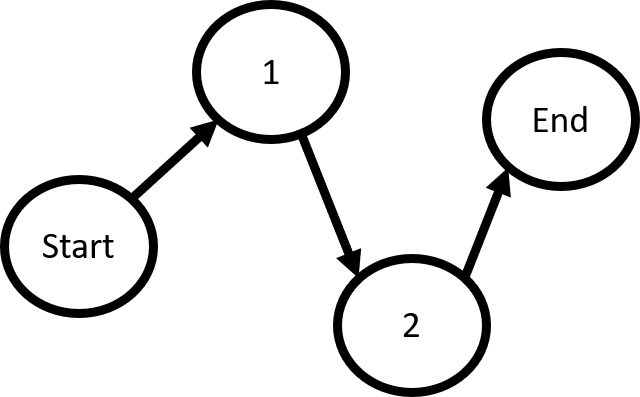 |
| --- |
| **Figure S1. Expected directed graph based on problem domain.** |

The graph has four nodes: start, 1, 2, and end. Each graph will have a start and end node. The other nodes represent events of interest in the problem being modeled. For example, the main text describes a model of HIV diagnostic testing guidelines in which these nodes represent HIV tests with or without a result (e.g., positive HIV screen, HIV nucleic acid test). The number of nodes will vary based on the problem. A self-referential edge will leave from and return to the same node. A bidirectional edge will transition back and forth between two nodes.

1. Assemble the observed data

| **Table S1. Observed data.** | | | |
| --- | --- | --- | --- |
| Patient Identifier | Node | Time stamp | Facility |
| 1 | 1 | 01/01/2000 | 1 |
| 1 | 2 | 01/10/2000 | 1 |
| 2 | 1 | 01/05/2000 | 1 |
| 2 | 2 | 01/06/2000 | 1 |
| 3 | 2 | 01/03/2000 | 2 |

The observed data consists of sequential observations made for each patient in the study population. The start and end nodes are not present in the observed data, but they are not explicitly observed. Although we have used the term “patient identifier” here, “sequence identifier” is an appropriate abstraction.

1. Create the observed graph

| **Figure S2. (A) Directed graph of observed transitions.** The thickness of the edge represents the count of the number of occurrences. **(B) Table of edges** | |
| --- | --- |
| (A)  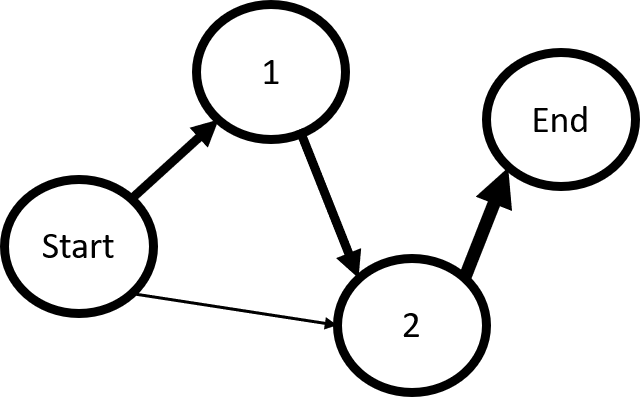 | (B)   \| Tail Node \| Head Node \| Count \| \| --- \| --- \| --- \| \| Start \| 1 \| 2 \| \| 1 \| 2 \| 2 \| \| 2 \| End \| 3 \| \| Start \| 2 \| 1 \| |

The observed data is converted into a graph. Edges are added for the start and end nodes at the beginning and end of each patient’s observations.

1. Compare the expected graph to the observed graph

| **Figure S3. (A) Comparison of expected and observed transitions**. Green denotes expected edges, while red denoted unexpected edges. **(B) Table of edges** | |
| --- | --- |
| (A)  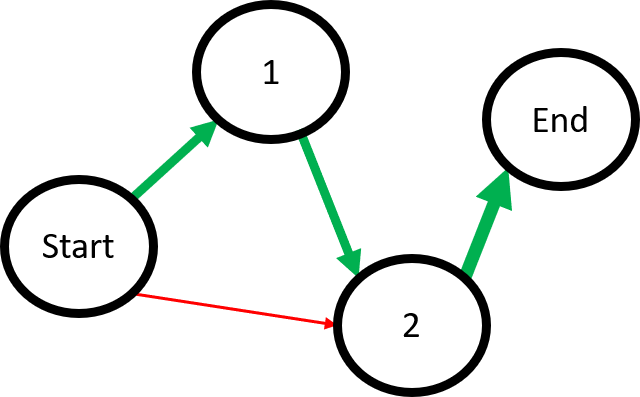 | (B)   \| Tail Node \| Head Node \| Count \| Expected \| \| --- \| --- \| --- \| --- \| \| Start \| 1 \| 2 \| Yes \| \| 1 \| 2 \| 2 \| Yes \| \| 2 \| End \| 3 \| Yes \| \| Start \| 2 \| 1 \| No \| |

1. Analysis of comparison between observed and expected graphs

| **Table S2. Summary table of comparison graph with additional metrics added (e.g., “Edge 1 Total”, “Edge 1 %”).** “Edge 1 Total” refers to the number of patients who experienced the “Tail Node” (e.g., 3 patients began at the “Tail Node” = “Start”). More metrics could be added to the table as needed to review the data (e.g., “…”). For example, the number of patients with each edge type could be added as a column. | | | | | | |
| --- | --- | --- | --- | --- | --- | --- |
| Tail Node | Head Node | Expected | Count | Edge 1 Total | Edge 1 % | *…* |
| Start | 1 | Yes | 2 | 3 | 66% |  |
| Start | 2 | No | 1 | 3 | 33% |  |
| 1 | 2 | Yes | 2 | 2 | 100% |  |
| 2 | End | Yes | 3 | 3 | 100% |  |

| **Table S3. Facility summary table of comparison graph.** In a healthcare system, the data may originate from multiple facilities, which may be split into separate lines to aide in review. | | | | | | |
| --- | --- | --- | --- | --- | --- | --- |
| Tail Node | Head Node | Expected | Count | Edge 1 Total  By Facility | Edge 1 % By Facility | Facility |
| Start | 1 | Yes | 2 | 2 | 100% | 1 |
| Start | 2 | No | 1 | 1 | 100% | 2 |
| 1 | 2 | Yes | 2 | 2 | 100% | 1 |
| 2 | End | Yes | 2 | 2 | 100% | 1 |
| 2 | End | Yes | 1 | 1 | 100% | 2 |

The comparison between the two graphs can be summarized by multiple tables. These tables can be stored in a workbook (e.g., Microsoft Excel) with different worksheets. Each worksheet represents a different view of the data (e.g., overall, by facility, by patient). The review of this data by a subject matter expert may identify promising targets for further review (e.g., manual validation).

## Example 2: Consideration of Absolute Time

This example, in contrast to the prior example, incorporates a time window to handle events that occur at near identical times. The problem under consideration dictates the length of the time window. We will choose 7 days. The computer code for these examples is available.^16^

1. Construct the expected graph based on the problem domain

| A  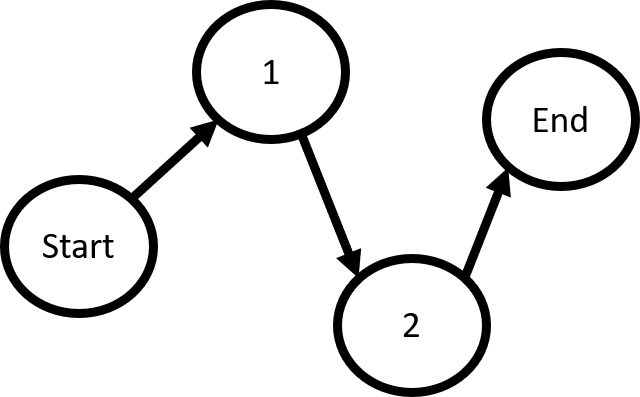 | B  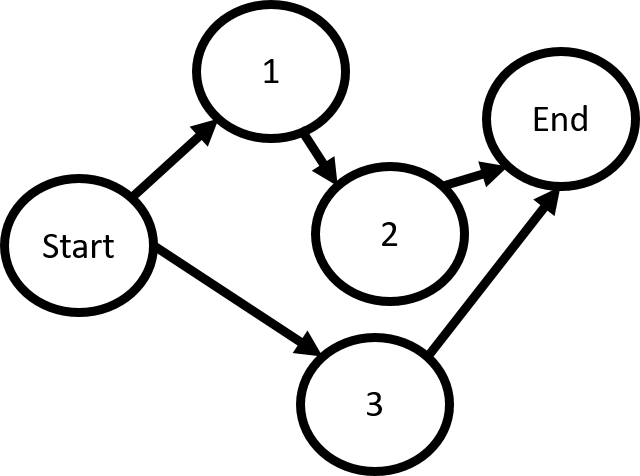 |
| --- | --- |
| **Figure S4. Expected directed graph based on problem domain. (A) Original graph without modifications for time. (B) Modification of the graph to account for a new node, “3”.** The node labeled “3” denotes the occurrence of events 1 and 2 within a given time window (e.g., 7 days). | |

1. Assemble the observed data. The node “3” does not exist in the original data (Table S4). Node “3” is added because nodes “1” and “2” occur within 7 days (01/05/2000 and 01/06/2000). The nodes “1” and “2” are combined into a new node “3” (Table S5).

| **Table S4. Observed data without time window.** | | | |
| --- | --- | --- | --- |
| Patient Identifier | Node | Time stamp | Facility |
| 1 | 1 | 01/01/2000 | 1 |
| 1 | 2 | 01/10/2000 | 1 |
| 2 | 1 | 01/05/2000 | 1 |
| 2 | 2 | 01/06/2000 | 1 |
| 3 | 2 | 01/03/2000 | 2 |

| **Table S5. Observed data with time window.** | | | |
| --- | --- | --- | --- |
| Patient Identifier | Node | Time stamp | Facility |
| 1 | 1 | 01/01/2000 | 1 |
| 1 | 2 | 01/10/2000 | 1 |
| 2 | 3 | 01/06/2000 | 1 |
| 3 | 2 | 01/03/2000 | 2 |

1. Create the observed graph

| **Figure S5. (A) Directed graph of observed transitions.** The thickness of the edge represents the count of the number of occurrences. **(B) Table of edges** | |
| --- | --- |
| (A)  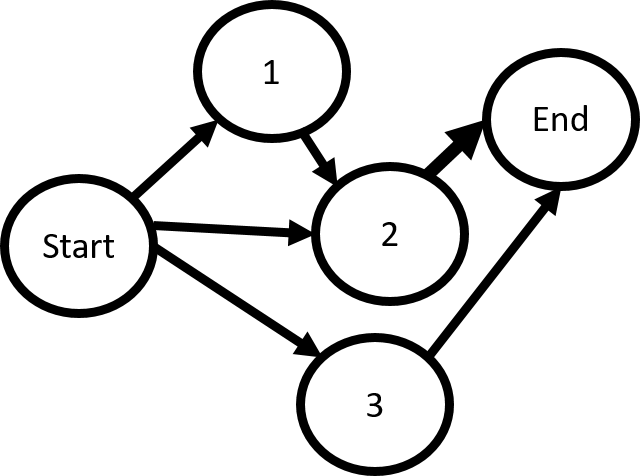 | (B)   \| Tail Node \| Head Node \| Count \| \| --- \| --- \| --- \| \| Start \| 1 \| 1 \| \| Start \| 2 \| 1 \| \| Start \| 3 \| 1 \| \| 1 \| 2 \| 1 \| \| 2 \| End \| 2 \| \| 3 \| End \| 1 \| |

1. Compare the expected graph to the observed graph. The graph in Figure S6A represents the comparison between the expected (Figure S4B) and observed (Figure S5A).

| **Figure S6. (A) Comparison of expected and observed transitions**. Green denotes expected edges, while red denoted unexpected edges. **(B) Table of edges** | |
| --- | --- |
| (A)  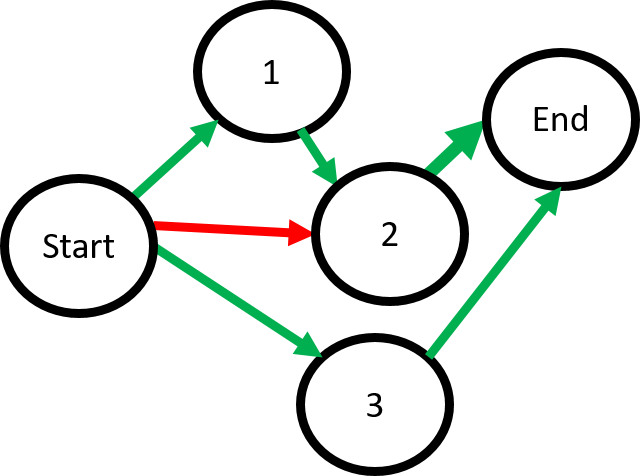 | (B)   \| Tail Node \| Head Node \| Count \| Expected \| \| --- \| --- \| --- \| --- \| \| Start \| 1 \| 1 \| Yes \| \| Start \| 2 \| 1 \| No \| \| Start \| 3 \| 1 \| Yes \| \| 1 \| 2 \| 1 \| Yes \| \| 2 \| End \| 2 \| Yes \| \| 3 \| End \| 1 \| Yes \| |

1. Analysis of comparison between observed and expected graphs

| **Table S6. Summary table of comparison graph with additional metrics added (e.g., “Edge 1 Total”, “Edge 1 %”).** “Edge 1 Total” refers to the number of patients who experienced the “Tail Node” (e.g., 3 patients began at the “Tail Node” = “Start”). More metrics could be added to the table as needed to review the data (e.g., “…”). For example, the number of patients with each edge type could be added as a column. | | | | | | |
| --- | --- | --- | --- | --- | --- | --- |
| Tail Node | Head Node | Expected | Count | Edge 1 Total | Edge 1 % | *…* |
| Start | 1 | Yes | 1 | 3 | 33% |  |
| Start | 2 | No | 1 | 3 | 33% |  |
| Start | 3 | Yes | 1 | 3 | 33% |  |
| 1 | 2 | Yes | 1 | 1 | 100% |  |
| 2 | End | Yes | 2 | 2 | 100% |  |
| 3 | End | Yes | 1 | 1 | 100% |  |

| **Table S7. Facility summary table of comparison graph.** In a healthcare system, the data may originate from multiple facilities, which may be split into separate lines to aide in review. | | | | | | |
| --- | --- | --- | --- | --- | --- | --- |
| Tail Node | Head Node | Expected | Count | Edge 1 Total | Edge 1 % | Facility |
| Start | 1 | Yes | 1 | 2 | 50% | 1 |
| Start | 2 | No | 1 | 1 | 100% | 2 |
| Start | 3 | Yes | 1 | 2 | 50% | 1 |
| 1 | 2 | Yes | 1 | 1 | 100% | 1 |
| 2 | End | Yes | 1 | 1 | 100% | 1 |
| 2 | End | Yes | 1 | 1 | 100% | 2 |
| 3 | End | Yes | 1 | 1 | 100% | 1 |

# Count of Possible Edges in a Graph

In this section, we will provide an upper limit on the number of edges allowed in the graph as defined in this application. The possible number of edges will increase as more nodes are added to a graph. This occurred in “Example 2: Consideration of Absolute Time” when node 3 was added to the graph. A graph constructed from actual data may not approach the upper limit of possible edges. But, review of these calculations should provide the reader with two critical insights: (1) The addition of more nodes to a graph is likely to increase the number of edges and complicate its interpretation. (2) Amongst the many possible edges, graphs can very effectively highlight the most critical edges, especially when the edges displayed in the graph are only shown when they reach a threshold number of occurrences (e.g., >1,000).

**Graph definition:** The graph we describe is directional, may contain loops, and has a start and end node. The start and end node cannot connect to each other with an edge. The head of an edge cannot point to the start node, and the tail of an edge cannot leave the end node. The start and end nodes cannot contain loops. With these considerations, the upper limit on the number of edges in this graph is given by Equation 1.

| **Equation 1: Upper limit on the number of directed edges in a fully connected multigraph with a start and end node (simplified equation).** x, number of nodes. x > 2. |
| --- |
| $x^{2}-2*x$ |

Equation 1 is derived from Equation 2. The terms in Equation 2 originate from Metcalfe’s law modified for a bidirectional graph (term 1), minus the edges that would point toward or away from the start and end nodes respectively (term 2), and with loops allowed for all nodes except the start and end nodes (term 3).

| **Equation 2: Upper limit on the number of directed edges in a fully connected multigraph with a start and end node (equation not simplified).** Metcalfe’s law is visible in the first term. x, number of nodes. x > 2. |
| --- |
| $\left( \frac{x*\left( x-1 \right)}{2} \right)*2-\left( 2*\left( x-2 \right)+2 \right)+\left( x-2 \right)$ |

When the method described in the section “Consideration of Absolute Time” is implemented, the number of nodes can dramatically increase (see Example 2 above). The number of possible nodes that may form is given by Equation 3.

| **Equation 3: Upper limit on the number of possible nodes when they are permitted to combine as described in the section titled “Consideration of Absolute Time”.** The start and end nodes (n=2; term 1) are added to (term 2) the summation of all combinations of the other nodes. x, number of nodes including the start and end nodes. x > 2. |
| --- |
| $2+\sum_{r=1}^{x-2} \binom{(x-2)}{r}=2+\sum_{r=1}^{x-2} \frac{(x-2)!}{r!*\left( (x-2)-r \right)!}$ |

**Example 1:** If the number of nodes in a graph is 3, including one start and end node, the maximum number of edges it contains is 3: Start 🡪 node 1, node 1 🡪 End, node 1 🡪 node 1 (loop). The nodes in the graph cannot combine, so Equation 3 does not change the result.

**Example 2:** If the number of nodes in a graph is 9, including start and end nodes, they can combine to form up to 129 nodes according to Equation 3. The maximum number of edges it contains is 16,383 according to Equation 1.

- Equations 3: 2 + (7 choose 1) + (7 choose 2) + (7 choose 3) + (7 choose 4) + (7 choose 5) + (7 choose 6) + (7 choose 7) = 2+7+21+35+35+21+7+1 = 129
- Equation 1: 129*129-2*129 = 16,383

Like this example, the graph used in the paper had 9 nodes: start, (-) screen, (+) screen, (-) confirm, (+) confirm, nucleic acid test (NAT), resistance, Western blot, end.

## SUPPLEMENTARY TABLES & FIGURES

| Table S8: Description of HIV Tests | | |
| --- | --- | --- |
| HIV Test | **Definition** | **Limitations** |
| Rapid HIV test | Rapid HIV tests can be performed outside of a clinical laboratory and provide results within minutes^1^ | Rapid HIV tests and HIV-1/2 antigen/antibody combination immunoassays could not be disambiguated |
| HIV-1/2 antigen/antibody combination immunoassay | HIV screening test performed by a clinical laboratory | The generation of the HIV screening test could not be determined (e.g., 3^rd^, 4^th^, 5^th^). |
| HIV-1/HIV-2 antibody differentiation immunoassay | HIV confirmation test performed by a clinical laboratory |  |
| HIV-1 nucleic acid test (NAT) | Alternatively referred to as HIV viral load. Indicated for diagnosis and prognosis |  |
| Western blot | This test is no longer recommended. |  |
| HIV resistance test | A heterogenous group of tests used to identify resistance mutations impacting antiretroviral treatment efficacy |  |
| ^1^<https://www.cdc.gov/mmwr/PDF/wk/mm5310.pdf> | | |
| 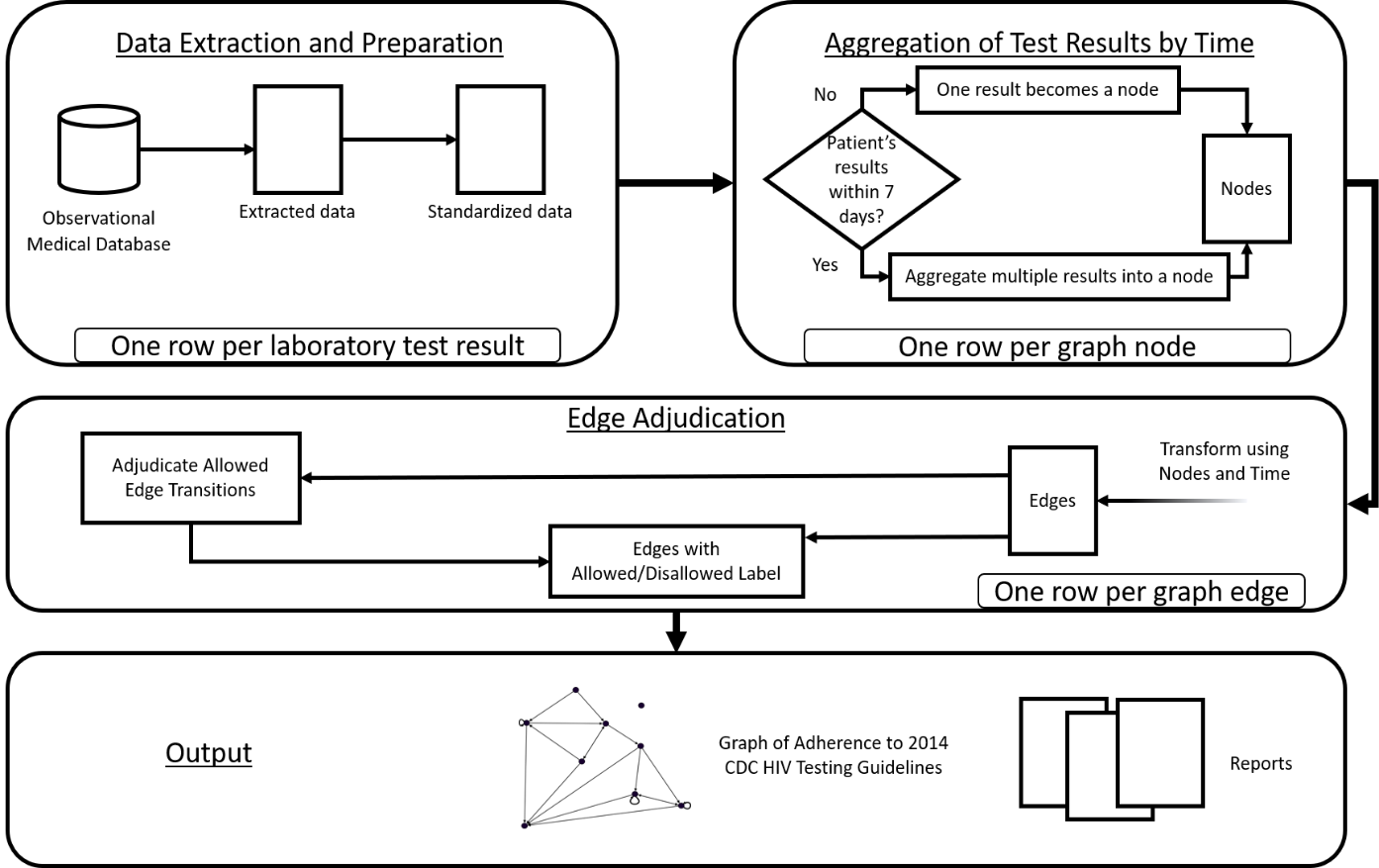 | | |
| Figure S7. Data flow from Input (i.e., Observational Medical Database) to Output (i.e., Graphs, Reports) | | |
