# Supplementary material for: Graphical analysis of guideline adherence to detect systemwide anomalies in HIV diagnostic testing
Source: PLoS One. 2022 Jul 1;17(7):e0270394. doi: 10.1371/journal.pone.0270394 (PMC9249187; doi:10.1371/journal.pone.0270394)
Supplement: Attachment — Submitted filename: Response to Reviewers.docx [file pone.0270394.s002.docx]

To the editor:

Thank you for addressing the delay incurred with this submission. We appreciate that it has been difficult to secure reviewers to evaluate the study.

We have reviewed the style requirements, amending the manuscript as necessary.

1. Changed headings to sentence case
2. Modified heading style 1, 2, and 3 to match the recommendations
3. Added a funding section: “Funding: The study had no designated funds, and the authors received no specific funding for this work. Therefore, funders had no role in study design, data collection and analysis, decision to publish, or preparation of the manuscript.”
4. Modified the figure references from “Fig.” to “Fig”.
5. Specified the data was identifiable when we received it. See line 142, “Nearly all facilities contributed identifiable data for the full duration of the study.” Note: Due to the sensitive nature of HIV and the VA data sharing policy, we are not permitted to place this data set in the public domain.

Reviewer #1: The authors propose to use graphical models to evaluate if guidelines are followed.

This is done by comparing the observed and guideline recommended graphs for HIV diagnostic testing. My comments are:

a) I think similar procedures are in place at many places. For example, when my group works with EHRs we perform similar procedures to check for data quality. Without user friendly software being available that allows easy implementation of the method is different settings, the impact of this is unclear.

Response a) We believe the reviewer has suggested that procedures similar to what we have described are “in place at many places”. We agree that there are similar processes for visual checking of data quality and that the availability of user-friendly software for easy access to these procedures is indeed lacking. But we believe that the packaging of the data quality checks with guidelines in a graphical form is novel, and we have made the software available in the public domain for others to use.

b) In EHRs it is pretty common to have entry errors. Did the manual chart review check for those?

Response b) Data entry errors are certainly common when manual processes are used. The dataset for this project was created by automated platforms used by clinical laboratories. Typographic errors are relatively rare with these automated platforms. (See https://pubmed.ncbi.nlm.nih.gov/28505339/.) We employed manual chart review to determine the clinical scenario in which the test was used.

c) Guidelines are not laws and are meant to guide but not completely dictate clinical decision making. Sometimes external circumstances justify not following the guidelines. Do the authors have any sense of how often the clinicians made a conscious decision not to follow the guidelines?

Response c) We agree that guidelines should not dictate the practice of medicine. We found that the use of HIV resistance tests instead of the HIV screen (Scenario 2) were 100% (n=50) nonadherent to guidelines and mistakes by these clinicians. In the discussion we mention a “simple intervention (e.g., phone call) to reverse course, such as with nonadherent HIV resistance tests (Figure 3).” We did indeed place a phone call to these providers, and they freely admitted they had mistakenly ordered the HIV resistance test instead of the HIV screen test. This was likely the reason for the drastic improvement in Figure 3.

d) It would be useful and interesting to have more discussion of the downstream effect of these checks. For example, more details on the intervention and how it was implemented in this setting would be helpful.

Response d) We do not have more details about the intervention. As described in the response above, the intervention consisted of us making providers aware of their erroneous orders over the phone. The extent of this intervention is described in the discussion. We also do not want to distract from the novelty of the paper, mainly the graphical methodology.

e) The authors state “The method does not involve advanced mathematics (e.g., algebra, calculus).” Can you give some examples of methods that require that?

Response e) Test utilization methods that involve advanced mathematics would include this paper written by one of the study authors (see https://www.ncbi.nlm.nih.gov/pmc/articles/PMC4355837/). The appendix of this paper contains an algebraic derivation.

Reviewer #2: This is a nice paper, clearly presented, good, generally reusable methodology. My only comment is that you miss an opportunity to show off your approach. Why not do the following:

1) Have Figure 1B show potential erroneous edges (or maybe anticipate the important ones and just include those, if there are too many possible permutations).

Response 1) We appreciate the kind comments from this reviewer, and we certainly do want to show off our approach! The reviewer has asked we show the erroneous edges in Figure 1B. These solid edges in this graph show patterns that adhere to guidelines. The dotted lines show nonadherent patterns.

2) Show the figure currently in 1B as a separate figure in the Results section and use line thickness to show frequency, as you mention in your introduction.

Response 2) We appreciate the suggestion, and we created Figure 1B as requested. As stated in the paper, we reviewed 8.790 million HIV tests with an estimated 20 thousand incorrect tests performed. The ratio of correct tests to incorrect tests makes the line thickness for the correctly performed tests much thicker than those of the incorrect tests. The line thickness of the incorrect tests is smaller and less noticeable to the reader. We want to draw the reader’s attention to the incorrect test ordering, because even though these tests make up a minority, they could lead to a missed diagnosis of HIV. We want to highlight this. For these reasons, we did not include the figure in the manuscript.

3) Add a new two-part figure to complement the current figure 3 that shows before and after, as weighted-edge graphs.

Response 3) Weighted-edge graphs use the line thickness to convey information about the frequency of the edge. We interpreted this question in a similar line of reasoning to the question above, which requested the use of line thickness, as in a weighted-edge graph. We came to a similar conclusion when deciding to use a weighted-edge graph in this situation. Because the number of incorrect tests makes a thinner line compared to the thicker line of the correctly performed tests, the reader’s attention is drawn away from the incorrectly performed tests. Even though we do not feel this suggestion is in the best interest of the manuscript, we appreciate the reviewer’s thoughtful critique.

It is not often I ask for a chance to re-review a paper (maybe never) but in this case I would be delighted to see a revised paper with the graphs as described above.
